# Detecting amino acid preference shifts with codon-level mutation-selection mixture models

**DOI:** 10.1186/s12862-019-1358-7

**Published:** 2019-02-26

**Authors:** S. Omar Kazmi, Nicolas Rodrigue

**Affiliations:** 1 Department of Biology, Carleton University, 1125 Colonel By Drive, Ottawa, K1S 5B6 Canada; 2 Institute of Biochemistry and School of Mathematics and Statistics, Carleton University, 1125 Colonel By Drive, Ottawa, K1S 5B6 Canada

**Keywords:** Substitution models, Monte Carlo methods, Mixture models

## Abstract

**Background:**

In recent years, increasing attention has been placed on the development of phylogeny-based statistical methodologies for uncovering site-specific changes in amino acid fitness profiles over time. The few available random-effects approaches, modelling across-site variation in amino acid profiles as random variables drawn from a statistical law, either lack a mechanistic codon-level formulation, or pose significant computational challenges.

**Results:**

Here, we bring together a few existing ideas to explore a simple and fast method based on a predefined finite mixture of amino acid profiles within a codon-level substitution model following the mutation-selection formulation. Our study is focused on the detection of site-specific shifts in amino acid profiles over a known sub-clade of a tree, using simulations with and without shifts over the sub-clade to study the properties of the method. Through modifications of the values of the amino acid profiles, our simulations show different levels of reliability under different forms of finite mixture models. Sites identified by our method in a real data set show obvious overlap with those identified using previous methods, with some notable differences.

**Conclusion:**

Overall, our results show that when a site-specific shift in amino acid profile is strongly pronounced, involving two clearly different sets of profiles, the method performs very well; but shifts between profiles that share many features are difficult to correctly identify, highlighting the challenging nature of the problem.

## Introduction

Detecting shifts in site-specific amino acid preferences across species or strains poses a number of technical challenges [1]. Some early approaches relied on information-theoretic calculations, performed on sequence alignments directly [2–4]. These methods assume that the molecular sequence of each species or strain in an alignment provides an independent set of observations across all sites. Quite often, however, the sequences of an alignment under analysis are in fact closely related to one another, and methods that fail to account for these relations are susceptible to numerous types of problems [5]. Indeed, the issue of non-independence of sequences in a sample is what drives the field of phylogeny-based methods of analysis [6, 7].

In keeping with this understanding, phylogenetic approaches that account for shifts in amino acid preferences have been explored, with perhaps the most sophisticated of these being the CAT-BP model [8]. Its name is meant as a shorthand for a model which, in effect, attempts a *CATegorization* across sites of amino acid profiles, with *Break-Points* along the tree that make changes to the amino acid profiles governing each site. Realizations of the substitution process are thus heterogeneous across the positions of an alignment, and along the branches of the phylogeny, with both types of heterogeneity inferred directly from the data. While the principles of the CAT-BP model are attractive, the time-heterogeneity of amino acid composition is modulated globally across all sites, which is a different perspective than one seeking to uncover particular sites having undergone changes in amino acid profiles. Moreover, the model is difficult to work with in practice, given the elaborate set of Monte Carlo operators involved in its implementation, and more work is needed to make the CAT-BP model tractable for larger data sets.

Roure and Phillipe [9] investigated an approach building solely on the CAT model [10], with a multi-stage system aimed at testing specific hypotheses about the time-heterogeneity of amino acid profiles across a few sub-clades of a phylogenetic tree. In their approach, a first Markov chain Monte Carlo (MCMC) run is conducted with the CAT model on a dataset of interest. On the basis of this MCMC, a finite set of profiles is constructed, which is meant to be an approximation of the distribution of amino acid profiles across sites. Using the so-defined empirical finite mixture model, a second set of MCMC runs is conducted, with each run taking place on a particular sub-set of taxa of the original data, in order to obtain an estimate of the posterior probability of a frequency profile given the data at the site. A post-treatment of this second set of MCMC runs is performed on the basis of site allocation probabilities in each sub-set of taxa, so as to compute the probability of identical profiles (PIP) across taxa sub-sets for each site. A low PIP signals a potential shift in amino acid preference across taxa sub-sets. Similar ideas have been explored by Rey et al. [11]. However, a strictly amino acid-level approach as used by Roure and Phillipe [9] and Rey et al. [11] relinquishes the mechanistic formulation [12] that allows for the analyses to be conducted directly on the protein-coding DNA sequence data, in a joint estimation of several features of the evolutionary process.

Codon-level approaches, relying on the mutation-selection framework [12, 13] have more recently been applied to the modelling of changes in amino acid profiles, either with site-specific-clade-specific maximum likelihood estimation [14–16], which may sometimes run the risk of over-parameterization [17]; with experimentally derived profiles [18], which are only applicable for few genes; or with hierarchical Bayesian methods such as the *differential selection profile* model [19], which intrinsically treat across-site variation as random-effects, and in this case account for across-time variation over specific clades of interest. Again, the computational challenges from the latter approach are significant, and the richness of the differential selection profile parameterization seems to leave it with low power [19].

Here, we adopt a method that combines some of the ideas proposed in Roure and Phillipe [9] with the mutation-selection approach described in Rodrigue et al. [20]. The method operates with a predefined finite mixture of amino acid profiles, which are introduced into the mutation-selection codon substitution framework [20, 21]. This modelling framework implicitly captures (non-synonymous) rate-heterogeneity as a consequence of its basic construction. For instance, if a codon site is allocated to a profile dominated by a single amino acid, it will have a very low non-synonymous rate (because a fixation factor included in the substitution matrix will approach 0 for any mutation away from the high-fitness amino acid); whereas another codon site, allocated to a profile with all amino acids being nearly equal in fitness would have a comparatively high non-synonymous rate (because the fixation factor will be close to 1 for all mutations); and altogether, given the mixture over a range of profiles, the model mechanistically leads to a high level of across-site rate heterogeneity. Similarly, the mutation-selection models we study here implicitly allow for the possibility of rate heterogeneity across lineages (or across sub-clades), through a mechanistic rationale whereby a codon site could shift from a profile, say, dominated by a particular amino acid (and thus with low non-synonymous rate) to a profile that is even across the twenty states (and thus of high non-synonymous rate). Through various shifts over a mixture of profiles, the model can capture a high level of rate-heterogeneity across a sub-clade of interest. In contrast to traditional modelling approaches, which attempt to capture rate heterogeneity phenomenologically (e.g., by fitting distributions of branch length multipliers [22, 23], through branch-site models [24, 25], or through Markov-modulated processes across branches [26]), mutation-selection models seek to parameterize the underlying causes of such rate heterogeneity. In this work, we allow different amino acid fitness profiles across sites, and over different parts of the tree. Specifically, MCMC is applied separately across different sub-sets of taxa from the data, corresponding to different parts of a phylogenetic tree. Using simulations in a realistic set of conditions, we study the identifiability, or strength of allocation, and PIP scores to evaluate the potential of our method. We find that the method has good power, with a reasonable false-positive rate, when the shifts in amino acid profiles correspond to marked differences in profiles. However, when the distinctiveness of profiles is low across the sub-tree of interest compared to the rest of the tree, the method can perform poorly. This is a common occurrence in real data settings and emphasizes that this problem is a challenging one. Our simulations illustrate one way of studying how the method’s performance changes for different levels of profile distinctiveness. Analysis on real avian- and human-host strains of Influenza shows results consistent with previous methods, with some notable exceptions.

## Methods

### Models and Monte Carlo sampling methods

The codon-level substitution model we use follows the mutation-selection framework [12, 13, 20]. At codon site *n*, the entries in the 61×61 matrix *Q*^(*n*)^, specifying the infinitesimal rate from codon *i* to codon *j*, are given as:

$$Q^{(n)}_{ij} = \mu_{ij} \frac{S^{(n)}_{ij}}{1-e^{-S^{(n)}_{ij}}}$$

where *μ*_*ij*_ controls the nucleotide-level mutational process ($\mu _{ij}=\rho _{i_{c} j_{c}}\varphi _{j_{c}}\phantom {\dot {i}\!}$, where *i*_*c*_ corresponds to an index of the nucleotide at position *c* (*c*=1,2, or 3) of codon *i*, and where (*ρ*_*ab*_)_1≤*a*,*b*≤4_ is a set of nucleotide exchangeabilities with the constraint $\sum _{1\le a< b\le 4} \rho _{ab}=1$, and *φ*_*a*_=(*φ*_*a*_)_1≤*a*≤4_ is a set of nucleotide propensities with the constraint $\sum ^{4}_{a=1}\varphi _{a}=1\phantom {\dot {i}\!}$) and $S^{(n)}_{ij}$ is the (scaled) selection coefficient associated with going from the amino acid encoded by *i* to that encoded by *j* at site *n* [20]. This selection coefficient is obtained from $S^{(n)}_{ij} = \ln \psi ^{(z_{n})}_{f(j)} - \ln \psi ^{(z_{n})}_{f(i)}\phantom {\dot {i}\!}$, where $\psi ^{(z_{n})}\phantom {\dot {i}\!}$ is the mixture component (amino acid profile) allocated to site *n*, $\phantom {\dot {i}\!}\ln \psi ^{(z_{n})}_{f(i)}$ is the (scaled) fitness of the amino acid encoded by codon *i* at that site, *f*(*i*) returns an index from 1 to 20 based on the amino acid encoded by codon *i*, and *z*_*n*_ is an auxiliary variable returning an index, from 1 to *K*, specifying the allocation of site *n* to component *k* (1≤*k*≤*K*).

In this work, our finite mixture models are based on two predetermined sets of amino acid profiles. First, we arbitrarily defined a set of profiles (which we denote “MutSelBC”) based loosely on a grouping of side chain biochemical properties:

- Small nonpolar: alanine, glycine, serine, threonine
- Aromatic: phenylalanine, tryptophan, tyrosine
- Nonpolar aliphatic: isoleucine, leucine, valine, methionine
- Polar positive: histidine, lysine, arginine
- Polar negative: aspartic acid, glutamic acid
- Polar neutral: asparagine, glutamine
- Proline
- Cysteine

This grouping into eight profiles was selected to have no overlap in amino acid residues, so as to construct the most biologically obvious shifts in amino acid preferences. We controlled the intensity of purifying selection against amino acids excluded from a group by distributing the probability mass of a profile mainly to the members of a group as explained in the opening of the results section.

As a second alternative, we used the C10, C20, C40, and C60 finite mixture profiles from Quang et al. [27], which were derived from an amino-acid-level maximum likelihood analysis of a large set of empirical data. As in Rodrigue et al. [20, 21], we refer to these models as MutSelC10, MutSelC20, MutSelC40, and MutSelC60.

Our Markov chain Monte Carlo sampler performs updates on *z* (*z*=(*z*_*n*_)_1≤*n*≤*N*_ where *N* is the number of codon sites), along with other parameters collectively denoted as *θ*, but we focus our description on the update mechanisms of the former, since the approaches we utilize are seldom discussed in the contexts of phylogenetic finite mixture models. We explore two sampling approaches. First, assuming an initial random allocation has been set, a Gibbs update for the allocation of a particular datum, denoted as *D*_*n*_, proceeds through the following steps: first, supposing that the datum allocation being subjected to the update is currently set to component *k*, i.e., *z*_*n*_=*k*, we decrease by 1 the count of the number of data columns affiliated to that component, denoted *η*_*k*_. Then, among the *K* possible components of the mixture, we draw a new *k*, and set *z*_*n*_=*k*, with probability ∝(*η*_*k*_+1)*p*(*D*_*n*_∣*θ*,*ψ*^(*k*)^). The auxiliary variable approach to our sampler is a form of *demarginalization* or *parameter expansion* [28], with respect to approaches that operate with weighted sum likelihood function at site *n*:

$$\begin{array}{@{}rcl@{}} p(D_{n} \mid \theta) = \sum_{k=1}^{K} w_{k} p\left(D_{n} \mid \theta, \psi^{(k)}\right),  \end{array} $$

where *w*=(*w*_*k*_)_1≤*k*≤*K*_ (with the constraint $\sum _{1\le k\le K}w_{k}=1$), is a weight vector, with *w*_*k*_ being the prior probability of a given site being allocated to component *k*. Updating as we do implicitly integrates over the weights, and is equivalent to having a flat Dirichlet on them [29]. Alternatively, we worked with a sampler that includes the weights, and draws a value *k* for site *n*, and sets *z*_*n*_=*k*, with a probability ∝*w*_*k*_*p*(*D*_*n*_∣*θ*,*ψ*^(*k*)^). This second sampling approach has the advantage of being paralellizable, since updating the allocation of one site does not rest on knowledge of the allocation states at other sites. Since both sampling methods produced very similar results, we worked with the paralellizable version given its greater computational efficiency.

### Probability of identical profiles

Using preset values for our mixture models, in conjunction with our MCMC sampling methods, allows for straightforward calculations of the probabilities of each site of an alignment belonging to each component of the mixture. Specifically, from a collection of draws of *z* from the posterior probability distribution obtained via MCMC, we calculate the probability of allocation of site *n* to component *k* as simply the proportion of draws where *z*_*n*_=*k* in our sample, which we denote as *p*^(*n*)^(*k*). Letting $z_{n}^{(m)}$ be the *m*th draw (from a total of *M*) from the posterior obtained by MCMC, the allocation probability is computed as

$$\begin{array}{@{}rcl@{}} p^{(n)}(k)= \frac{1}{M}\sum_{m=1}^{M}\delta_{mnk}, \end{array} $$

where

$$\begin{array}{@{}rcl@{}} \delta_{mnk}= \left\{\begin{array}{ll} 1, \text{if} {z}_{n}^{(m)} = k,\\ 0, \text{otherwise}. \end{array}\right. \end{array} $$

The same procedure can be applied for an analysis where the allocation of site *n* is (potentially) different in two parts of the tree, in this case giving us the probability of allocation of site *n* to component *k* in the human-host sub-tree $p_{\text {hu}}^{(n)}(k)$, and that of the remaining avian-host part of the tree $p_{\text {av}}^{(n)}(k)$. Doing so amounts to assuming complete independence of the human sub-clade from the rest of the tree. In other words, we make the crude assumption that the branch length connecting this sub-clade is of infinite length. In this latter context, the probability of identical profiles [9] at site *n* is calculated as follows:

$${PIP}_{n}=\sum\limits_{k=1}^{K} p_{\text{hu}}^{(n)}(k) \times p_{\text{av}}^{(n)}(k). $$

Note that *PIP*_*n*_=1 for a site with 100% probability of allocation to the same profile across the two parts of the tree and *PIP*_*n*_=0 if the allocation is entirely to different profiles.

The PIP can therefore be used to sort sites of interest when seeking to uncover those which have undergone a shift in amino acid preferences. As an arbitrary cutoff, we study the sites having PIP from 0 to 0.05 (see below), but a more permissive approach could use a higher PIP cutoff value.

### Real data

**Fig. 1:**
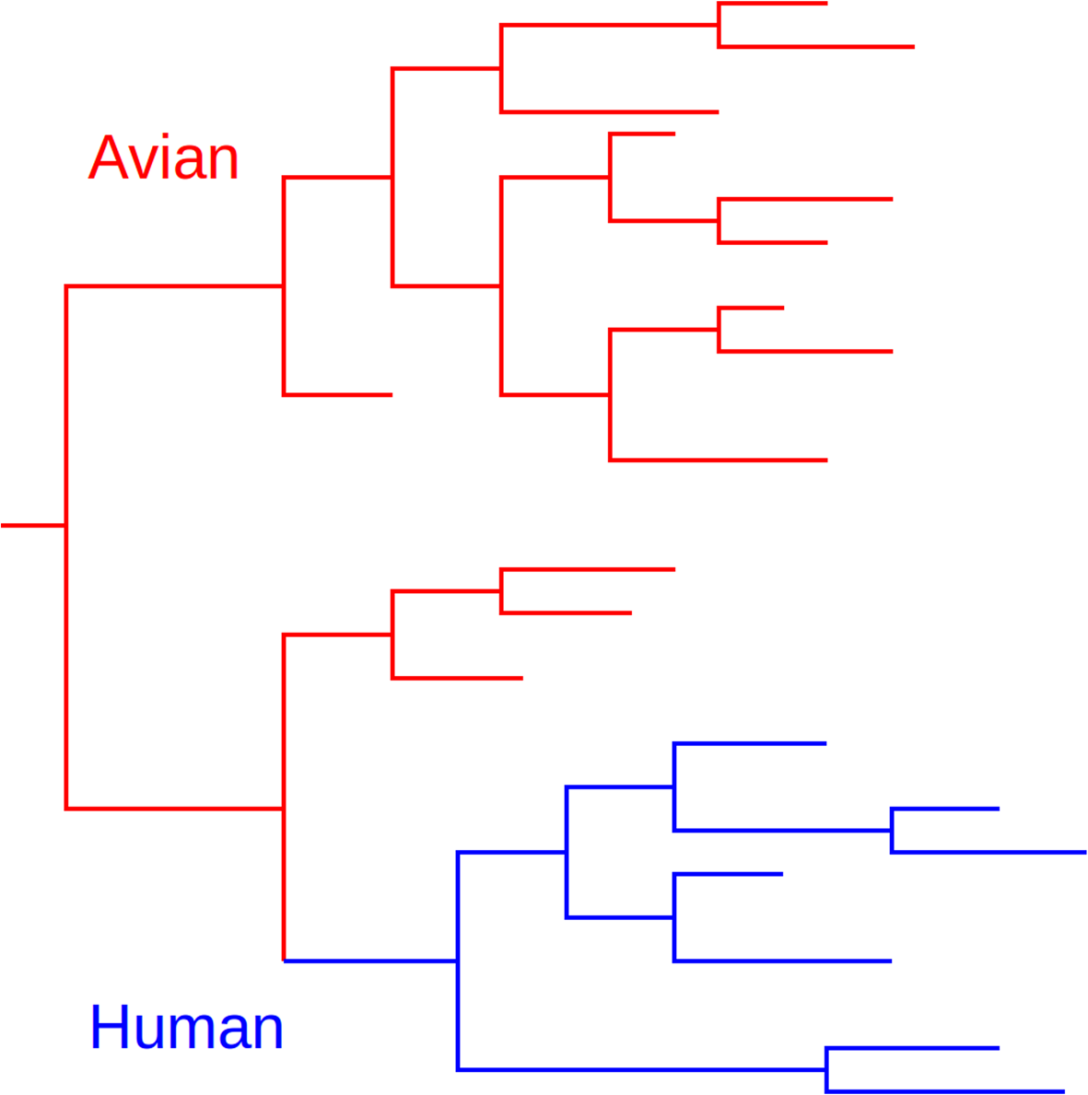
Sketch of Influenza PB2 phylogenetic tree, with monophyletic human-host clade

We used the Influenza PB2 gene alignment assembled by Tamuri et al. [30], comprised of 321 avian-host and 80 human-host strains. The reference tree topology was also taken from Tamuri et al. [30] and was invariant throughout the analysis. This tree is structured such that human-host strains are monophyletic, as sketched in Fig. 1. We are therefore focused on detecting shifts that may have occurred in human-host strains, following the transfer from avian hosts.

### Simulations

We simulated data using the posterior mean nucleotide-level parameter values (closely matching those reported in previous studies [17, 30]) and branch length values obtained when running the MutSelC60 model [20], combined with several different amino acid profiles, as described above. When analyzing this simulated data, nucleotide-level parameters were re-sampled from the posterior distribution, as were branch lengths and amino acid profile allocations. The profiles themselves were kept fixed, as was the tree topology. Some simulations consisted of running the same substitution process over the entire tree (comprising both human- and avian-host strains), which can be viewed as the *control* simulations, whereas others used different amino acid profiles in the human-host strains sub-tree than in the remainder of the (avian-host strains) tree.

We explored simulations under several sets of amino acid profiles. The first set, which we denote “MutSelBC”, is arbitrarily defined, and based loosely on side chain biochemical properties (see above). These profiles were selected to have no overlap in the dominant amino acid residues, such that biologically clear preference shifts at a given position would be represented as profile shifts coinciding with the host transition [30].

These simulations and calculations were conducted in a modified version of PhyloBayes-MPI 1.7 [31, 32], which outputs allocation probabilities for MCMC runs under finite mixture models (available).

## Results and discussion

### Simulation data

**Fig. 2:**
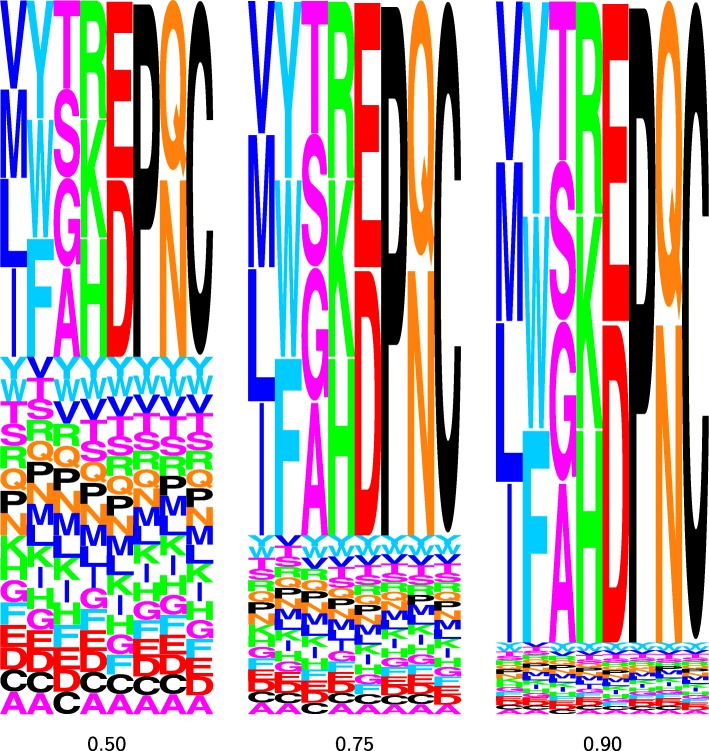
Logoplots of MutSelBC profile sets with four different “peakedness” values, indicating probability mass of the chosen amino acids in each profile. For each profile set, the probability of the profile-specific amino acid is 50%, 75%, and 90% respectively

Figure 2 depicts three versions of the eight MutSelBC profiles that we used for our first series of simulations. In these logoplots, the height of each letter in the column represents the probability mass for that amino acid residue in the profile. These three sets of profiles show a gradient in what we refer to as *peakedness*. We use this term to refer to the probability mass that we distribute equally to the amino acids of a profile group, with the complement being distributed equally to the remaining amino acids, not part of that group. The first set of profiles shows a 50% peakedness value (leftmost panel of Fig. 2). Thus, in the first profile in this set, valine, methionine, leucine and isoleucine together have a 50% probability mass, with the other 50% equally distributed to all other amino acids; the second profile in the set has 50% distributed evenly to tyrosine, tryptophan, and phenylalanine, with the 17 other amino acids splitting the remaining 50%, and so on. The two other panels of Fig. 2 show profiles with dominant amino acids sharing a probability mass of 75% and 90%. The peakedness parameter provides a crude means of controlling the intensity of the constraint for the amino acids of a group. Note that the peakedness only applies when constructing profiles, and is not a parameter that comes into play during inference.

**Fig. 3:**
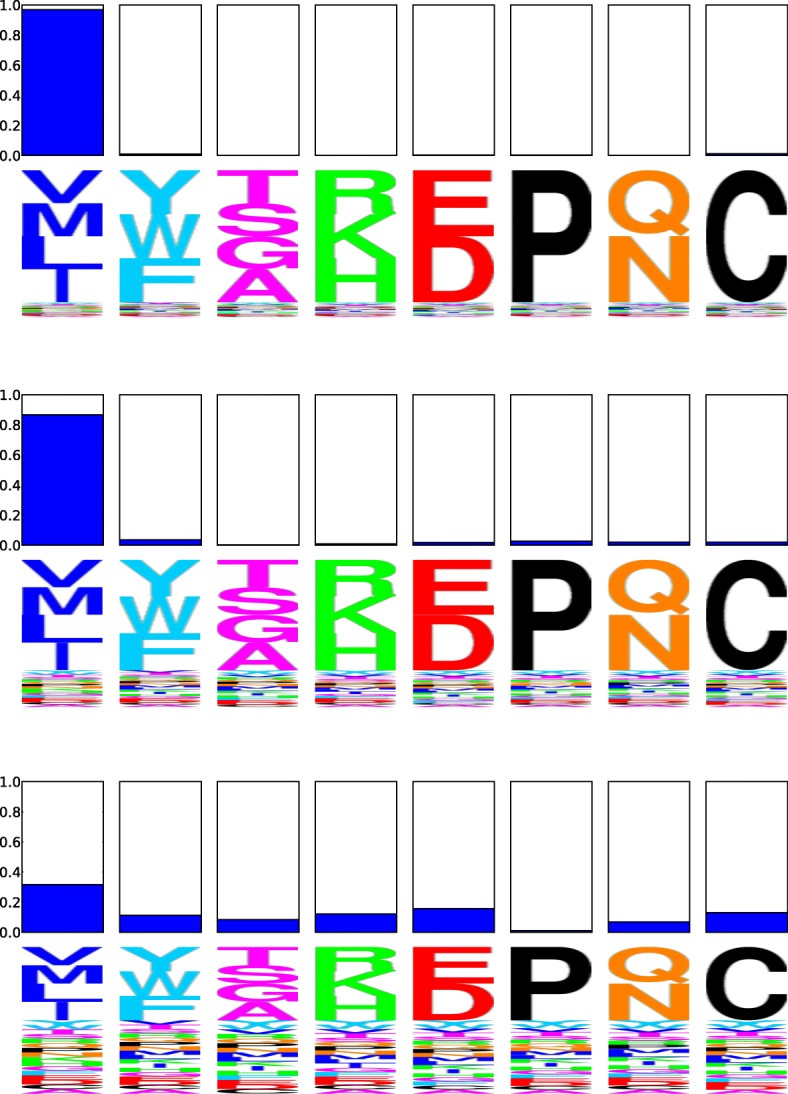
Allocation probability logoplots for the same codon site using MutSelBC profiles with 90% (top), 75% (middle) and 50% (bottom) “peakedness” values. For each logoplot, the solid bar indicates the allocation probability to a given profile at that site, and the letters underneath indicate the profile. The letters are scaled to indicate the probability of that amino acid at the site in that profile

The effect of the peakedness of the simulation profiles on the subsequent analyses can be seen in the logoplot in Fig. 3. This figure displays the allocation probability of a particular site, simulated with the first profile at 90%, 75% and 50% peakedness. These simulations respectively amount to what we could characterize as a relatively strong, moderate, and weak selection constraint for nonpolar aliphatic residues at that site. As the peakedness decreases, the allocation probability to the profile used to simulate can be seen to decrease. In other words, when the selection constraint for a particular group of amino acids is weak, so is the allocation probability.

**Fig. 4:**
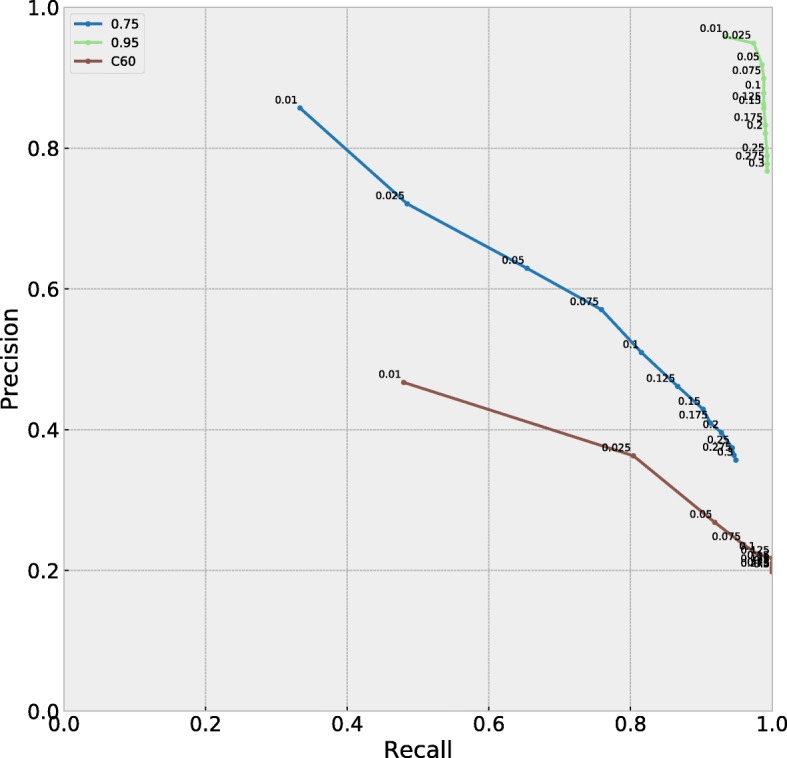
Precision-recall plots for MutSelBC profiles with peakedness 0.75 and 0.95, and MutSelC60

A precision-recall plot (Fig. 4) shows that the best PIP threshold across different profile sets was between 0.025 and 0.075, although barely perceptible graphically. We therefore chose 0.05 as a preliminary threshold for our study.

**Fig. 5:**
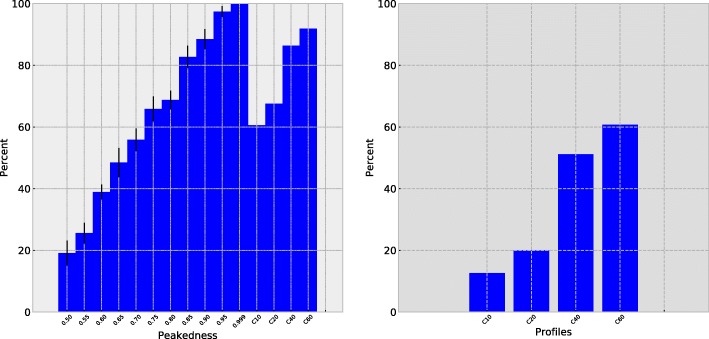
True positive left), and false positive (right) detection of preference shifts, by peakedness value, for simulated data using MutSelBC, MutSelC10, MutSelC20, MutSelC40 and MutSelC60 profiles

Figure 5 summarizes the broader implications of profile peakedness on the simulation results. Alignments were simulated as before, with a peakedness ranging from 0.50 to 0.95. For each alignment, 80 of the 759 codon sites were evolved under a different profile regime in the human-host sub-tree than in the rest of the tree, to simulate a distinct preference shift at those sites. The remaining sites were evolved under the same profiles for the entire tree. We then analyzed these alignments, evaluating the probability of identical profiles (PIP) at each site across the human-host sub-tree and the rest of the tree.

It can be seen that the true-positive detection rate, that is, the ability to correctly identify the sites that were evolved with different amino acid profiles across the two groups by having a low PIP score, increased dramatically as a function of the peakedness of the profiles (Fig. 5, left panel). A peakedness of 0.95 identified 87.3% of the embedded profile shifts. In other words, the approach is effective in detecting amino acid profiles which are markedly different in the sub-tree of interest. However, with a peakedness of 0.5, the true-positive detection rate is less than 20%, indicating that if the strength of constraint on a sub-set of amino acids is not sufficiently strong, the method will perform poorly.

False positive detection (Fig. 5, right panel), where PIP was low despite there not being an embedded shift, showed best performance at 0.95 peakedness (2.05%). Again, at this level of peakedness, the profiles are fairly rigid and there is little or no overlap of amino acids across the set of profiles.

Simulation analyses for MutSelC10, MutSelC20, MutSelC40, and MutSelC60 models showed considerably higher false positive rates compared to the MutSelBC models, with MutSelC10 being the lowest at 12.5% and MutSelC60 the highest at 60.7%. True positive rates were generally lower than with MutSelBC models, ranging from 60.6% for MutSelC10 to 91.8% for MutSelC60. This could be because of lower allocation probability to these profiles in general, as compared to MutSelBC. However, in many cases it is likely due to multiple profiles having similar amino acid compositions, while differing slightly in the proportions.

**Fig. 6:**
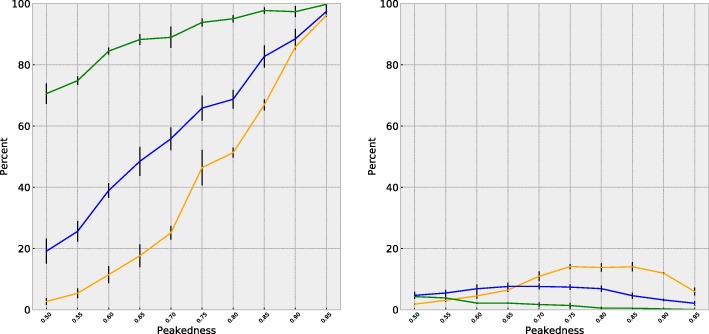
True positive (left), and false positive (right) detection of preference shifts using MutSelBC profiles in trees with the same topology but branch lengths altered by a factor of 0.1 (yellow), 1 (blue) and 10 (green)

In order to investigate the effect of varying amounts of evolutionary signal within data, we performed another set of simulations. We constructed a scenario that mimics a low-information content in the alignment, and another that mimics a high-information content; this was accomplished by taking the original tree used for our simulations and multiplying all branch lengths by a factor of 0.1 and 10, respectively for low- and high-information content in artificial data sets. As expected, data sets simulated with high-information content led to a better overall performance of the method, whereas those with low-information content led to a poorer performance. This can be seen in Fig. 6: in the left panel, we see that when branch lengths are one-tenth of the original (yellow line), that true positive detection is markedly lower in the same peakedness profiles; conversely, multiplying the branch lengths by ten (green line) notably increases the rate of true positive detection. However, even in the low-information context, profiles with a peakedness of 0.80 or higher perform better than chance in the detection of true positives. False positives (right panel) are not markedly altered by information content, remaining under 20% for all peakedness profiles.

**Fig. 7:**
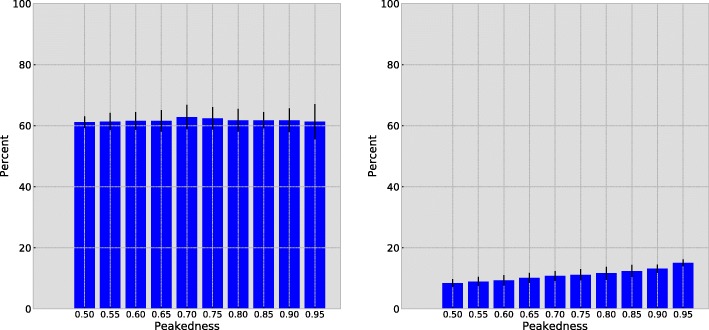
True positive (left), and false positive (right) detection of preference shifts for data sets simulated with MutSelC60 profiles but analyzed with MutSelBC profiles, by peakedness of MutSelBC profile

We conducted additional simulation studies where data was simulated under the MutSelC60 profiles and analyzed with the different (artificial) MutSelBC profiles, over a range of peakedness (Fig. 7). Results were not strongly differentiated: a shift from one MutSelC60 profile to another often does not amount to a biophysical or functional shift, and is thus not registered as a relevant shift by the different MutSelBC profiles, as shown below.

**Fig. 8:**
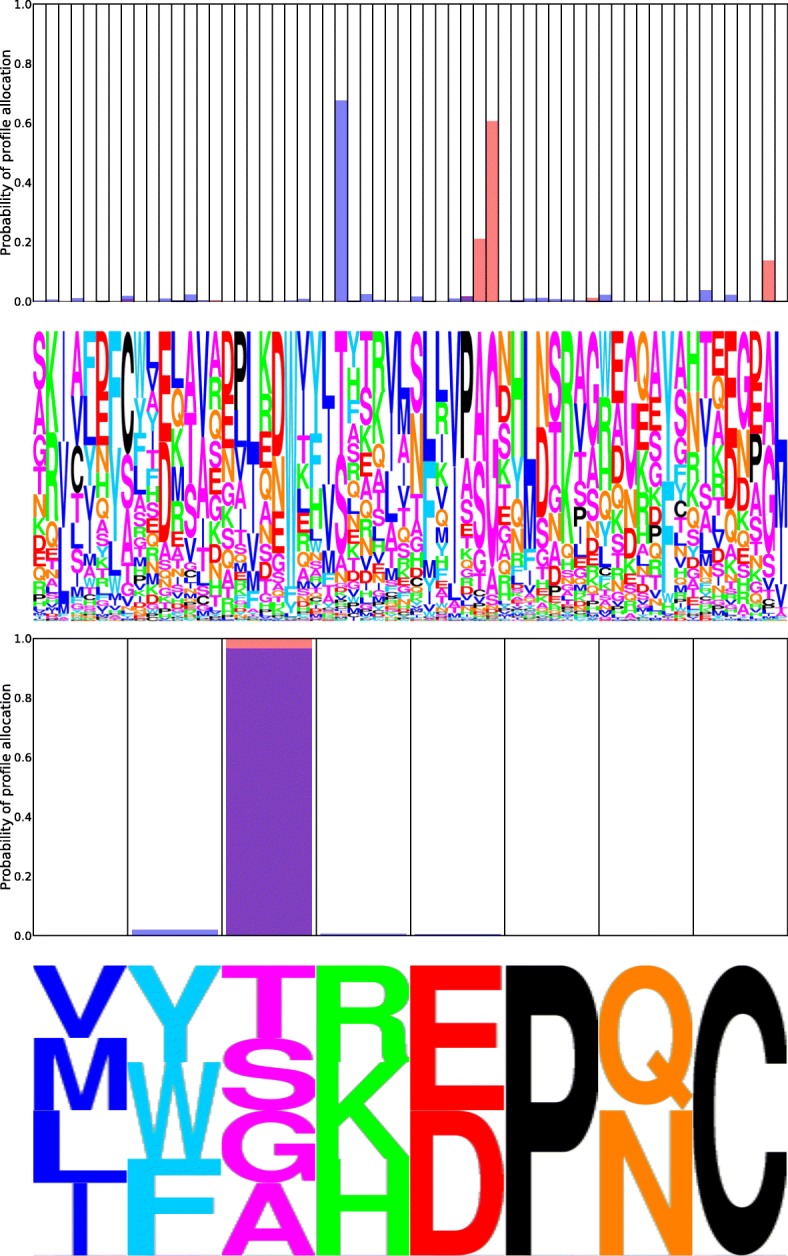
Allocation probability logoplot using MutSelC60 (top) and MutSelBC (bottom) profiles, illustrating a false positive in simulated data under MutSelC60. Red is the simulated avian-host alignment, and blue is the simulated human-host alignment, which did not have an embedded preference shift. Purple indicates overlapping profile allocation in both clades

Figure 8 is a demonstration of a potential false positive. It shows an apparent preference shift in the sub-clade of interest, whereas the simulation did not, in fact, include a shift at that site. The allocation probability in the human-host sub-tree is displayed in blue, and the allocation probability in the remaining avian-host strains is displayed in red. Closer examination of the profile allocations in the MutSelC60 case indicates that the apparent shift may be an artifact of the ambiguity of the profiles. In the avian-host strains, the site appears to allocate primarily to three different profiles, but all three of these profiles show preference for small nonpolar amino acids (ASTG). In the human-host sub-tree, the site almost entirely allocated to a single profile, different from those of the avian-host strains. Yet, this profile also shows preference for small nonpolar side chains (AST). There may be little functional difference in residues at the site, but different weighting of the same residues in different profiles gives a false positive. The MutSelBC profiles at bottom, which have a single profile for small nonpolar side chains (TSGA), show almost no difference in profile allocation between the two groups.

### Real data

**Fig. 9:**
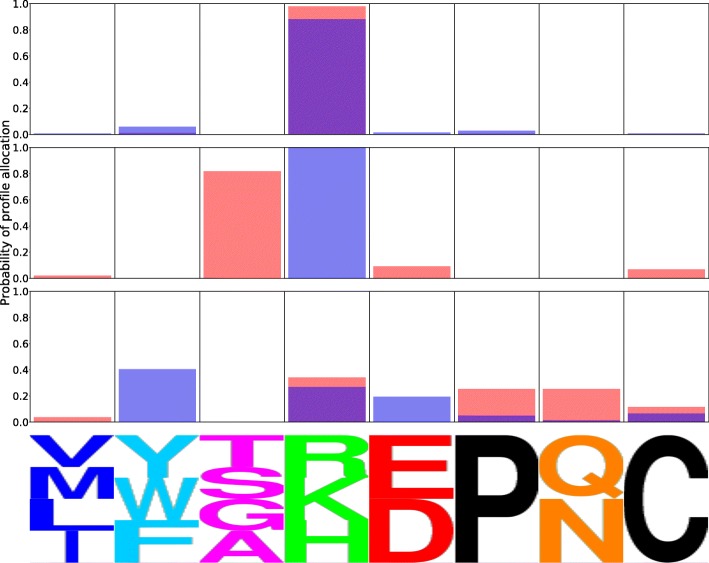
Allocation probability logoplots in avian (red) and human (blue) strains of Influenza PB2. Purple indicates overlapping profile allocation in both clades. At top is a strongly identified non-shift with a PIP of 0.865. At middle is a strongly identified shift, with a PIP of 0.00. At bottom, the low PIP of 0.115 does not indicate a preference shift, as site is weakly allocated to a number of profiles in both clades

Figure 9 shows allocation to MutSelBC profiles for sites with high and low PIP scores. In the top panel, the avian and human groups allocated strongly to the same profiles (PIP = 0.865), while the middle panel shows the case where both clades allocated completely to different profiles (PIP = 0.00). The bottom panel has a relatively low PIP score (0.115), but this is due to weak allocation to any profile, in both groups, rather than the result of a clear preference shift at that site.

Table 1 shows PIP scores for sites using MutSelC60 and MutSelBC models. Sites that were identified in Finkelstein et al. [3] as having undergone a shift in amino acid composition between human- and avian-host strains are also listed, along with the euclidean distance of their amino acid frequency vectors. Sites identified in Tamuri et al. [30] as displaying a preference shift between human and avian clades are also listed.

**Table 1:** Codon sites with preference shifts detected with MutSelC60 and MutSelBC profiles, and sites identified in previous studies ([3] and [30])

Site	PIP (MutSelC60)	PIP (MutSelBC)	Finkelstein	Tamuri	Avian	Human
44	0.0582	0.2155	0.966	YES	A((S))	S((L))((A))
64	0.148	**0.0170**	0.954		M(I)((T))	T((M))((I))
76	**0.00346**	**0**			T((M))((K))((I))((A))	T
81	**0.0165**	**0**			T((I))((A))	M(V)((T))((I))
102	0.166	**0.0000160**			N((S))((K))	N
105	**0.00535**	**0**		YES	T((A))	V(M)((T))((L))((I))
106	0.0184	1			T((A))	T(A)
107	**0.0116**	**0.00637**			S((N))((G))	S(N)
109	0.272	**0.0005**			V((I))	V(I)
122	0.0708	**0.00608**			V((M))((I))((A))	V((I))((F))
199	**0.0278**	1	0.997	YES	A*	S
249	0.445	**0**			E*	E
271	**0.0154**	0.974	0.958		T((I))((A))	A((T))
292	**0.0127**	**0.0265**			I(V)((T))((M))	T((I))
338	**0.0124**	**0**			V((I))((A))	V(I)
377	**0.00150**	0.0655			A*	A((V))((T))((E))
471	**0.00383**	0.0845			T((I))((A))	T((P))((I))((A))
475	0.69	1	0.994	YES	L*	M
493	**0.00257**	0.91		YES	R*	R((K))((G))
522	0.0516	**0.008**			Q*	Q((H))
524	0.168	**0.00547**			T((M))((I))	T((I))
559	**0.00799**	0.26			T((M))((I))((A))	T(A)((S))((N))((I))
567	0.0852	**0.216**	0.977		D((N))((E))	N((D))
569	0.175	1		YES	T*	T(A)((S))
588	**0.00283**	**0.00609**	0.971		A((V))((T))	I((V))((A))
591	0.265	**0.001**			Q((L))	Q
613	0.0776	0.115		YES	V((A))	T((V))((I))((A))
627	**0.0111**	**0**	0.977	YES	E((K))	K((R))
661	**0.00126**	0.967		YES	A((V))((T))	T((V))((A))
674	**0.00644**	0.0555	0.969		A((S))	T((P))((I))((A))
676	0.0165	0.242			T((I))((A))	I(T)
682	0.372	**0**		YES	G*	G(S)((N))
684	**0.0596**	0.999		YES	A((T))	S(A)
702	0.552	1	0.955	YES	K((R))	R((K))
711	0.17	**0**			N((S))	N
715	0.0916	**0.00584**			N((S))	N((T))
740	**0.00239**	**0**		YES	D*	D((N))
754	**0.0125**	**0.0335**			I*	I((S))((F))

Sites with a preference shift, defined as a PIP below the detection threshold of 0.05, are in bold. Amino acids have no parentheses if their frequencies are greater than 0.5, one set of parentheses if between 0.1 and 0.5, and two sets if between 0.01 and 0.1. An asterisk on a single amino acid indicates that other amino acids are present at a frequency of less than 0.01

Tamuri et al. [30]’s methodology in measuring the magnitude of the preference shifts makes it difficult to compare findings, but several cases exist where the shift as detected by Tamuri et al. [30] were to a functionally similar profile in MutSelBC. We illustrate several examples below.

Site 44 was allocated to alanine-dominant profiles in the avian-host strains and serine-dominant profiles in human-host strains, albeit with very weak allocation in the human-host clade. Tamuri identified an alanine preference in the avian-host strains and a leucine preference in human-host, but both had a strong secondary preference for serine.

Site 475 allocated strongly to leucine-dominant profiles in the avian-host strains, and methionine-dominant in human-host strains. Tamuri found similar results, with avian-host strains preferring leucine with a secondary preference for methionine, while human-host strains preferred methionine only.

Site 569 strongly allocated to threonine-dominant profiles in the avian-host strains, while the human-host clade was spread between threonine, alanine, serine and glycine-dominant profiles. Tamuri identified a preference for threonine (secondary alanine) in avian-host strains, and alanine (secondary serine) in human-host strains.

Site 613 showed weak allocation in both clades, with avian-host strains favouring valine-dominant profiles and human-host strains split between valine, methionine and leucine. Tamuri identified a preference for valine with secondary alanine and isoleucine for avian-host strains, while human-host strains has a preference for threonine, with secondary isoleucine and alanine.

Site 702 strongly allocated to lysine-dominant profiles in the avian-host strains and arginine-dominant profiles in human-host strains. Tamuri identified a preference for as lysine with secondary arginine in avian-host strains, and arginine in human-host strains.

As can be seen, instances where neither MutSelC60 or MutSelBC profiles agreed with Tamuri et al. [30] were largely due to the biochemical similarity of the residues involved, which caused allocation to similar or identical profiles, or due to Tamuri et al. [30] identifying a change in proportion of preference to the same amino acids between groups, which was not considered a functional shift under our finite mixture models.

## Conclusions and future directions

The idea behind our mixture modelling approach is this: each codon site of the alignment is considered to have been generated under one particular component (profile) from the mixture. Our MCMC system allows for the calculation of the posterior probabilities of a site having been generated by each of the possible components. If a site has an equal probability of being affiliated to one of two profiles in both the human-host clade and the rest of the data, than the model is indeed saying that, given the evidence at hand, there is a 0.5 probability that the site evolved under the same profile in the entire data set (by chance, there is a 0.5 probability of picking the same profile in both sub-sets of sequences). Such cases simply reflect the uncertainty of inference. Moreover, if a site has a nearly equal probability of having evolved under each of the MutSelC60 profiles in the human-host sub-clade, and likewise in the avian-host set, the PIP would be very low; given the evidence at hand, the model is saying that it is quite unlikely that the same profile acted in both sub-sets of sequences. For the purposes of our study, such latter cases are not of direct interest. Rather, our focus is on those sites exhibiting strong evidence of a profile shift. This amounts to focusing on sites that have reasonably strong allocation in both parts of the tree, but where those allocations are different. One way to guide this focus would be to take the entropy of the allocation probability vector of a site into account. This entropy quantifies the strength of the constraint at that site, and the phylogenetic signal available in the sequence alignment.

Overall, we can see that finite mixture models are capable of detecting preference shifts in simulated viral sequence alignments, especially where the profile shifts are highly pronounced. This is the case for MutSelBC profiles with a high peakedness value: inferences become progressively less powerful as shifts become less prominent. However, these profiles are arbitrarily defined and relatively crude, with equal probability mass given to all residues in a profile.

MutSelC60 profiles, which are more objectively constructed from empirical data, show a middle ground in effectiveness of preference shift detection. However, one drawback of using this mixture model is the allocation of sites to similar profiles, registering as a profile shift and resulting in false positives, as detailed in the discussion for Table 1 above. This raises the question of whether profile shifts between biologically similar residues truly represent adaptive shifts. After all, empirical amino acid matrices, such as LG [e.g., 33], are based on the rationale that some pairs of amino acids are highly exchangeable, and may be nearly equivalent in fitness. Alternatively, it may be that statistically significant shifts may have low biological significance.

The MutSelBC model is blind to these types of shifts. For example, A199S is detected as a host shift marker by MutSelC60, Finklestein et al. [3] and Tamuri et al. [30], but is contained in the same profile and considered strictly equivalent by MutSelBC.

Important improvements could be realized by using empirical profiles constructed within the codon mutation-selection context, rather than MutSelC60 profiles, which were originally derived in an amino-acid replacement context. We could extend this further by defining empirical codon profiles so as to detect shifts in codon usage [34]. For example, the original MutSelC60 profiles could be mapped onto three sets of 61-element codon profiles: one with the bulk of the probability mass on high-usage codons, one with emphasis on low-usage codons, and one with equal weighting on all degenerate codons. This would allow us to simultaneously investigate site-specific heterogeneity in both amino acid preference and codon usage bias.

We could also investigate a model which modulates the efficacy of selection across different parts of the tree. In the mutation-selection framework, this can be accomplished by introducing a parameter corresponding to an effective population size [35]. In fact, such a modelling approach, modulating the role of selection in the substitution process, amounts to a simpler objective than seeking to uncover bona-fide changes in amino acid preferences, but could more compactly capture time-heterogeneity in amino acid composition.

Finally, another modelling direction could aim to recognize the possibility that a site could be allocated to the same profile in different sub-trees, say one dominated by I, L, M and V, while having very different overall "flux" across the high-fitness amino acids. The underlying ideas for such models have been preliminarily explored by Rodrigue & Lartillot [36], in an approach that modulates overall non-synonymous rates multiplicatively with both $S_{ij}^{(n)}/\left (1-e^{-S_{ij}^{(n)}}\right)$ and *ω*_∗_, in order to detect genes in which the non-synonymous rates are higher than expected under the nearly-neutral mutation-selection modelling formulation. Bloom [37] has also explored this modelling strategy in a site-specific fashion. By extending the approach to accommodate different *ω*_∗_ values across sub-trees, one could detect variation in the non-synonymous flux even when the underlying amino acid fitness profile is the same in both sub-trees. If this modelling extension could then be combined with the ideas in the present study, one could hope to jointly detect shifts in non-synonymous flux and shifts in amino acid fitness profiles.

## Abbreviations

CAT: CATegorization substitution model using per-site amino acid profile classes
CAT-BP: CAT model which includes breakpoints along the tree that govern profile changes
LG: Le & Gascuel’s amino acid substitution model [33]
MCMC: Markov-chain Monte Carlo
MutSelBC: Mutation-selection profiles based on biochemical side chain groupings
MutSelC60: Mutation-selection profiles based on C60 profiles in Quang et al. [27]
PIP: Probability of identical profile
